# School Dangers

**Published:** 1876-04

**Authors:** 


### SCHOOL DANGERS.

MANY girls and boys of promise, the great hope of life to yearning parents, are sacrificed, every year, to the cupidity of sordid, stupid, or reckless school-teachers, aided and abetted by the contemptible vanity of the thoughtless parents themselves. We regard public examinations and school-exhibitions a cheat and a sham, in three cases out of four. It is done for the benefit and behoof of the teacher, and to the irreparable injury of the scholar; while the poor dolt of a parent has not sense enough to see through it. We hope never to see a child of ours competitor for any prize or station at school.

Not long since, a gentleman of wealth, from the East, consulted us in behalf of an only child, a daughter of seventeen, at school. She was expected to complete her studies, at an academy, in two months. Already she had been preparing for an examination, for some weeks. The report was, that she was so much "interested" in her studies, that she barely allowed herself necessary sleep; that she always ate in haste, and went to her books immediately after her meals. She had all the symptoms of a commencing decline, and she was determined to "keep up" until the close of the session. Those two months seemed to us an interminable age ahead. We felt as if she ought to have been hurried out of the school-room, without an hour's delay, and driven out among the beautiful hills of her own New England, and scarcely allowed time out of the saddle to take her meals: we felt as if she ought to have been compelled to eat most of her meals on horseback. But the gratification which was to result to her from a successful examination, outweighed all considerations of the happiness of healthful youth. We declined giving special advice while she was at school. We have no doubt that the reaction which will take place after the examination will, with her previous condition, send her to an early grave—as it has done in multitudes of similar cases before. Parents ought to remember, that reviewing studies for an examination is for the glorification of the teacher, without any commensurate advantage to the scholar.

A young lady, the hope of a widowed mother, and both poor, wrote, only in June last, that she was at school, preparing herself as a teacher, with a view to support herself and mother, by obtaining a position in the school of which she was then only a scholar; but, in order to do that, it was necessary that her examination should entitle her to a diploma. How long and how hard she had been striving, we do not know; but the struggle had been so severe, the tension so great and continued, that she writes: "A weakness and drowsiness has come over me, from which I cannot arouse myself, and causes me almost to despair of recovery. Mere talking is a weariness. It seems as if I shall never feel wide awake again. I feel as if I could sleep forever. This sleepiness is experienced, not only at noon and at night, but also in the early morning. Having always ranked first in my classes at school, I have endeavored, the present year, to maintain my position; but I feel that my health is not equal to the task. It seems that the faculties of my mind are not what they once were, especially my memory. The time is drawing near, when the diplomas will be awarded to our class. The apprehension of a failure, on my part, weighs heavily on my mind; and fail I must, unless I can be aroused from my stupid state. The very efforts I make to keep myself awake in the day-time, often make me sick at heart."

Here is the case of a young brain stimulated to sheer exhaustion, while all the powers of life were failing with it. Out upon it, we say. Let the barbarous customs of the school-room be abolished; and let parents and teachers understand, that education can be so conducted as to make it a self-buoyant process, from the commencement of the alphabet to its successful close. Really competent teachers can make it a delight, instead of a burden and a bore—can make it the meat and drink of those who learn. These are practical teachers, and deserve treble salaries, with the respect and thanks of all the humane.

